# Control charts for measurement error models

**DOI:** 10.1007/s10182-022-00462-8

**Published:** 2022-10-05

**Authors:** Vasyl Golosnoy, Benno Hildebrandt, Steffen Köhler, Wolfgang Schmid, Miriam Isabel Seifert

**Affiliations:** 1 Faculty of Management and Economics, Ruhr University Bochum, Universitätsstrasse 150, 44801 Bochum, Germany; 2 Chair of Statistics, European University Viadrina, Frankfurt (Oder), Germany

**Keywords:** Statistical process control, Measurement error, Control charts, Volatility modeling, C22, C44, C58

## Abstract

We consider a linear measurement error model (MEM) with AR(1) process in the state equation which is widely used in applied research. This MEM could be equivalently re-written as ARMA(1,1) process, where the MA(1) parameter is related to the variance of measurement errors. As the MA(1) parameter is of essential importance for these linear MEMs, it is of much relevance to provide instruments for online monitoring in order to detect its possible changes. In this paper we develop control charts for online detection of such changes, i.e., from AR(1) to ARMA(1,1) and vice versa, as soon as they occur. For this purpose, we elaborate on both cumulative sum (CUSUM) and exponentially weighted moving average (EWMA) control charts and investigate their performance in a Monte Carlo simulation study. The empirical illustration of our approach is conducted based on time series of daily realized volatilities.

## Introduction

The class of measurement error models (MEMs) is of much importance in numerous empirical settings (Durbin and Koopman 2009; Hamilton 1994a). A standard MEM is a system of linear equations, where state equations describe dynamics of latent variables and measurement (observation) equations relate observable and latent variables via measurement errors. In this paper we consider popular linear MEMs which are bivariate linear state-space systems with only one observation equation, whereby the single state equation is an AR(1) process with iid innovations (cf. Hamilton 1994; Brockwell and Davis 2009). Under stationarity assumption, such MEM processes can exhibit a broad variety of autoregressive dynamics. Its essential characteristic is the ratio of the state innovation variance to the measurement error variance which determines the usefulness of a MEM representation for a particular application compared to a simple AR(1) alternative (cf. Kim and Nelson 1999; Tsay 2010; Bollerslev et al. 2016).

In this paper we focus on sequential (online) monitoring of possible changes in this variance ratio, which—to the best of our knowledge—has not been done in the current literature up to now. Our primer aim is to detect a change of the MEM to AR(1) or vice versa as soon as it occurs. For these purposes, we re-write the MEM under consideration equivalently as an ARMA(1,1) specification. In case of negligible measurement errors, ARMA(1,1) reduces to AR(1) model, so that our task is to detect alterations in the MA(1) part. Hence, we show that monitoring of the MEMs is equivalent to monitoring of changes from ARMA(1,1) to AR(1) and vice versa. Sequential monitoring of parameter changes in autoregressive models has been considered already in the early papers of Schmid (1997), Lu and Reynolds (2001); a comprehensive literature review is given by Okhrin and Schmid (2008) who discuss various aspects of monitoring changes in parameters of linear time series models. The literature dealing with the sequential monitoring of ARMA model parameters, however, is mostly concentrated on detection of changes in the means, see e.g., Jiang et al. (2000), Rabyk and Schmid (2016), Lazariv and Schmid (2019), Golosnoy and Seifert (2021), with a remarkable exception of Rosolowski and Schmid (2006) who elaborate tools for monitoring of the whole autocovariance function of a stationary ARMA(1,1) process.

For online monitoring changes in the MA(1) parameter, we apply control charts which are the instruments (decision rules) borrowed from statistical process control for the purpose to detect changes in the parameters of the process of interest as soon as they occur (cf. Montgomery 2013). In particular, we consider both exponentially weighted moving average (EWMA) and cumulative sum (CUSUM) control charts which are the most popular monitoring procedures in practice. The control statistics suitable for our monitoring task are derived based on the ideas of the statistical testing approach recently developed by Golosnoy et al. (2021) for the class of linear state space models. We investigate the detecting ability of these control charts for MA(1) parameter changes in an extensive Monte Carlo simulation study. We find that the EWMA charts with the smoothing (memory) parameter $\lambda =0.01$ provides the quickest detection for the zero-state average run length, whereas $\lambda =0.1$ for the conditional steady-state average run length criterion. These findings are in line with the results in Morais et al. (2015). Moreover, we provide an empirical illustration by monitoring daily realized volatility models for the major world indices of financial stock markets. The timing of signals from both EMWA and CUSUM charts allows the following economic interpretations: the AR(1) model appears to be more suitable for the crisis periods on financial markets with a high speed of information transfer, where the ARMA(1,1) suits better for the (comparatively) calm periods.

The rest of the paper is organized as follows. In Sect. 2 we introduce a MEM with AR(1) process in the state equation, and show that it could be equivalently written as an ARMA(1,1) process. Hence, monitoring for changes in the MEM is equivalent to monitoring for changes of the MA(1) parameter. We also introduce the control statistic which is suitable for our monitoring purposes. In Sect. 3 we propose both CUSUM and EWMA control charts for sequential (online) monitoring of changes in the MA(1) parameter. The detection performance of the control chart is evaluated in the Monte Carlo simulation study in Sect. 4. In Sect. 5 we provide an empirical application of the proposed control charts for monitoring the process of daily log realized volatility, as well as discuss directions for further research. Section 6 concludes the paper, whereas the proofs are placed in the Appendix.

## The measurement error model

Assume the following common measurement error model (MEM) representation for the de-meaned observable variable $y_t$:

1 $$\begin{aligned} y_t= \,& {} s_t + u_t, \quad \qquad ~ u_t\sim \mathcal {N}_{\text{ iid }} (0,\sigma ^2_u), \qquad \sigma ^2_u>0, \end{aligned}$$

2 $$\begin{aligned} s_t= \,& {} \phi s_{t-1} + \epsilon _t, \qquad \epsilon _t\sim \mathcal {N}_{\text{ iid }} (0,\sigma ^2_{\epsilon }), \qquad \sigma ^2_{\epsilon }>0, \end{aligned}$$

where the latent state variable $s_t$ exhibits an AR(1) dynamics so that we remain within the class of Markov processes; $u_t$ is the Gaussian measurement error, $\epsilon _t$ is the Gaussian innovation error with $u_t$ independent on $\epsilon _s$ for all *t*, *s*. Eq. (1) is referred to as the measurement (observation) equation and (2) is known as the state equation. The MEM stationarity condition is given by $|\phi |<1$, hereafter we focus on $\phi \in (0,1)$ (without loss of generality) which is the case in the majority of applications.

The essential characteristic of this MEM is the variance ratio $\sigma ^2_u/\sigma ^2_{\epsilon }$. In case when $\sigma ^2_u/\sigma ^2_{\epsilon } \rightarrow 0$ the measurement error gets negligible and the system boils down to an AR(1) equation. In case when $\sigma ^2_u$ is not negligible, one should apply the MEM. For a given data generating process our research question is whether a MEM is indeed required for modeling $y_t$-dynamics or the measurement error is negligible so that a simple AR(1) model could be used? This issue is of much empirical relevance because AR(1) models are much easier to handle compared to MEMs.

For the purposes of our analysis we re-write the MEM in Eqs. (1)–(2) in the form of an equivalent ARMA(1,1) representation which parametrization is provided in the next proposition.

### Proposition 1

Let $y_t$ follow a MEM, parameterized in (1) and (2) with an AR(1) parameter $\phi \in (0,1)$ and Gaussian iid innovations $u_t$ and $\epsilon _t$, with $u_t$ independent on $\epsilon _s$ for all *t*, *s*. Then the distribution of the MEM process given in (1) and (2) is equivalent to the distribution of an ARMA(1,1) process given by

3 $$\begin{aligned} y_t=\phi y_{t-1} + a_t + \theta a_{t-1},\qquad a_t\sim \mathcal {N}_{\text{ iid }}(0,\sigma ^2_a), \end{aligned}$$

with the parameters $\theta \in (-1,0)$ and $\sigma ^2_a>0$ which are given as the following functions of the MEM parameters

4 $$\begin{aligned} \sigma ^2_a= & {} -\phi \sigma ^2_u/\theta , \end{aligned}$$

5 $$\begin{aligned} \theta= & {} - \frac{\sigma ^2_{\epsilon }+\sigma ^2_u(1+\phi ^2)}{2\phi \sigma ^2_u} + \sqrt{\left[ \frac{\sigma ^2_{\epsilon }+\sigma ^2_u(1+\phi ^2)}{2\phi \sigma ^2_u}\right] ^2-1}. \end{aligned}$$

Hence, in case of $\sigma ^2_u\rightarrow 0$ it holds that $\theta \rightarrow 0$ and $\sigma ^2_a=\sigma ^2_{\epsilon }$, i.e., ARMA(1,1) reduces to AR(1) model.

As we are interested in monitoring whether the MA(1) part of the MEM is of relevance, we define the variable $x_t$ in an intermediate step by removing the AR(1) part:

6 $$\begin{aligned} x_t:=y_t-\phi y_{t-1} =\epsilon _t+u_t-\phi u_{t-1}=a_t + \theta a_{t-1}. \end{aligned}$$

Since $x_t$ is an MA(1) process, its autocovariance function is then given as

7 $$\begin{aligned} {{\,\mathrm{Var}\,}}(x_t)=\, & {} \sigma ^2_{\epsilon }+\sigma ^2_u(1+\phi ^2)=\sigma ^2_a \left( 1+\theta ^2\right) ,\nonumber \\ {{\,\mathrm{Cov}\,}}(x_t,x_{t-1})= & {} -\sigma ^2_u\phi =\,\sigma ^2_a \theta ,\nonumber \\ {{\,\mathrm{Cov}\,}}(x_t,x_{t-\ell })=\, & {} 0 \qquad \text{ for } \quad \ell \ge 2. \end{aligned}$$

In order to differentiate between AR(1) and ARMA(1,1) one should monitor for possible shifts in the MA(1) parameter $\theta$ which is of pivotal for our study. As we observe in (7), the MA(1) parameter $\theta$ influences linearly the covariance ${{\,\mathrm{Cov}\,}}(x_t,x_{t-1})$. For this reason we introduce another variable $v_t$ defined as

8 $$\begin{aligned} v_t:= \frac{x_tx_{t-1}}{\sigma ^2_a}\,. \end{aligned}$$

Next, by making use of the ideas and the results in Golosnoy et al. (2021) we derive the moments of $v_t$ which is given in the following proposition.

### Proposition 2

For the ARMA(1,1) representation of the MEM model given in (3) with the innovations $a_t\sim \; \mathcal {N}_{\text{ iid }}(0,\sigma ^2_a)$, the moments of $v_t=\,x_tx_{t-1}/\sigma ^2_a$ are given by

9 $$\begin{aligned} {{\,\mathrm{E}\,}}(v_t)= \,& {} \theta , \end{aligned}$$

10 $$\begin{aligned} {{\,\mathrm{Var}\,}}(v_t)=\, & {} 1+3\theta ^2+\theta ^4,\end{aligned}$$

11 $$\begin{aligned} {{\,\mathrm{Cov}\,}}(v_t,v_{t-1})= \,& {} \theta ^2, \qquad \text{ and } \qquad {{\,\mathrm{Cov}\,}}(v_t, v_{t-\ell }) =\, 0, \quad \text{ for } \quad \ell \ge 2. \end{aligned}$$

As $\theta \in (-1,0)$ it holds that ${{\,\mathrm{Var}\,}}(v_t)\in (0,5)$ and ${{\,\mathrm{Corr}\,}}(v_t,v_{t-1})\in (0,1/5)$.

Hence, due to ${{\,\mathrm{E}\,}}(v_t)= \theta$, monitoring changes in the mean of $v_t$ is equivalent to monitoring changes in the MA(1) parameter $\theta$. We exploit this property in the next section by applying the popular control charts for monitoring $\{v_t\}$-process in order to make online detection of changes in its mean.

## Control charts for MEMs

The majority of papers dealing with the online monitoring of parameter changes in ARMA(1,1) models and their extensions are focused on the detection of changes in the means (cf. Jiang et al. 2000; Bodnar and Schmid 2007; Golosnoy et al. 2012; Rabyk and Schmid 2016; Lazariv and Schmid 2019; Golosnoy and Seifert 2021). A prominent exception from this literature stand is the paper of Rosolowski and Schmid (2006) where several multivariate EWMA control charts are applied for a quite general task of monitoring changes in both mean and autocovariances of a stationary and invertable ARMA(1,1) process.

Compared to the general approach of Rosolowski and Schmid (2006), in this paper we pursue a more specific task of online monitoring for possible changes in the MA(1) parameter $\theta$. In general, we define the in-control value of the MA(1) parameter as $\theta =\theta _0$, whereas the out-of-control value is $\theta =\theta _1$, with either $\theta _1>\theta _0$ or $\theta _1<\theta _0$. A signal could be used as an indicator to judge whether the MEM should be re-calibrated. The particular shifts (special cases) we are eager to detect are, firstly, from AR(1) to ARMA(1,1) which implies a change from $\theta _0=0$ to some $\theta _1\in (-1,0)$; secondly, shifts from ARMA(1,1) to the direction of AR(1) which implies a change from $\theta =\theta _0$ for a given $\theta _0\in (-1,0)$ to $\theta =\theta _1>\theta _0$. In our empirical application we have also to cover the case with the in-control $\theta =\theta _0\in (-1,0)$ and the alternative $\theta =\theta _1\in (-1,0)$ such that $\theta _1<\theta _0$. Hence, the direction of shifts is known under the null hypothesis of no change (the in-control state), so that one-sided control charts are suitable. Next we introduce modified CUSUM- and EWMA-type control charts (cf. Okhrin and Schmid 2008) for the purpose of monitoring mean shifts in the (weakly) autocorrelated process $\{v_t\}$ defined in (8).

### CUSUM charts

The CUSUM charts are well-known monitoring procedures which exhibit some optimality detection properties. The control statistic of the upper-sided CUSUM control chart for detecting increases in MA(1) parameter $\theta _0$ is given by

$$\begin{aligned} S_t^+= & {} \max \{0, S_{t-1}^+ + (v_t-\theta _0) - \delta \}, \end{aligned}$$

whereby the CUSUM parameter $\delta$ is recommended to be selected as the half of the shift to detect:

12 $$\begin{aligned} \delta = \frac{1}{2} \,\Big |{{\,\mathrm{E}\,}}_{H_1}(v_t) - {{\,\mathrm{E}\,}}_{H_0}(v_t)\Big |= \frac{1}{2}\,\big |\theta _1 - \theta _0\big |. \end{aligned}$$

The run length of the upper CUSUM control chart is given by

$$\begin{aligned} RL^{+}= & {} \inf \{t\in \mathbb {N} \;| \, S_t^+ \, > \, h^+_{\delta } {{\,\mathrm{Var}}}_{0}(v_t)^{1/2}\}, \end{aligned}$$

with the in-control variance of $v_t$ given as ${{\,\mathrm{Var}}}_{0}(v_t)=1+3\theta _0^2+\theta _0^4$. The upper control limit $h^+_{\delta }$ is selected such that it provides a pre-defined in-control average run length (ARL). We use the headstart feature which is also known (cf. Lucas and Crosier 1982) as the fast initial response (FIR), and set the starting value as $S^+_0=h^+_{\delta } {{\,\mathrm{Var}}}_{0}(v_t)^{1/2}/2$.

The lower-sided CUSUM chart for detecting decreases in $\theta _0\in (-1,0)$ is defined by analogy via the statistic

$$\begin{aligned} S_t^-= & {} \min \{0, S_{t-1}^- + (v_t-\theta _0) + \delta \}, \end{aligned}$$

with the run length of the lower CUSUM chart given by

$$\begin{aligned} RL^{-}= & {} \inf \{t\in \mathbb {N} \;| \, S_t^- < h^-_{\delta } {{\,\mathrm{Var}}}_{0}(v_t)^{1/2}\}, \end{aligned}$$

where $h^-_{\delta }$ is the lower control limit.

### EWMA charts

The EWMA control charts are very popular in practice because of their simple design and good detecting properties. The EWMA control statistic $Z_t$ for monitoring mean changes in $\{v_t\}$-process is given by

$$\begin{aligned}&Z_t=\lambda (v_t-\theta _0) + (1-\lambda )Z_{t-1}=(1-\lambda )^t Z_0+\lambda \sum _{j=0}^{t-1}(1-\lambda )^j (v_{t-j}-\theta _0), \\&\quad Z_0=0, \, t\ge 1, \end{aligned}$$

with a smoothing parameter $\lambda \in (0,1]$. The value $\lambda =1$ leads to the no-memory Shewhart control chart.

The run lengths of the upper and lower EWMA control charts are given as

$$\begin{aligned} RL^+= & {} \inf \{t\in \mathbb {N} \; | \; Z_t>h^{+}_{\lambda } {{\,\mathrm{Var}}}_0(Z_t)^{1/2}\},\\ RL^-= & {} \inf \{t\in \mathbb {N} \; | \; Z_t<h^-_\lambda {{\,\mathrm{Var}}}_0(Z_t)^{1/2}\}, \end{aligned}$$

where ${{\,\mathrm{Var}}}_0(Z_t)$ is the in-control variance of statistic $Z_t$. Its exact expression is given by

13 $$\begin{aligned} {{\,\mathrm{Var}}}_{0}(Z_t)=\frac{\lambda }{2-\lambda }\left[ (1-(1-\lambda )^{2t})(1+3\theta _0^2+\theta _0^4) +2(1-\lambda )(1-(1-\lambda )^{2(t-1)})\theta _0^2\right] , \end{aligned}$$

with the derivation provided in the Appendix. For $t\rightarrow \infty$, the exact variance converges to the limit value

14 $$\begin{aligned} \underset{t\rightarrow \infty }{\lim }{{\,\mathrm{Var}}}_{0}(Z_t)= \frac{\lambda }{2-\lambda }\left[ (1+3\theta _0^2+\theta _0^4) +2(1-\lambda )\theta _0^2\right] . \end{aligned}$$

Further in our analysis we use the exact variance of the EWMA statistic given in (13).

## Monte Carlo simulations

In this section we evaluate the ability of the CUSUM and EWMA charts to detect changes in the MA(1) parameter $\theta$. For this purpose we investigate the performance of the CUSUM and EWMA control schemes in a Monte Carlo simulation study. In particular, we simulate $x_t$ directly as an MA(1) process:

$$\begin{aligned} x_t={\left\{ \begin{array}{ll} \theta _0a_{t-1}+a_t,&{}\text{ for }\ t<1\\ \theta _1a_{t-1}+a_t,&{}\text{ for }\ t\ge 1 \end{array}\right. }\qquad \text{ with } \qquad a_t \sim \, \mathcal {N}_{\text{ iid }}(0,1). \end{aligned}$$

The in-control value of the MA parameter is denoted by $\theta _0$. A change occurs at $t=1$ such that the MA parameter shifts to its out-of-control value $\theta _1$. We investigate two out-of-control settings which are mostly relevant for our analysis, namely the situations:

(i) $\theta _0=0,\ \theta _1=-1/2$, i.e., shift from AR(1) to ARMA(1,1), an upper-side control chart applicable;
(ii) $\theta _0=-1/2,\ \theta _1=0$, i.e., shift from ARMA(1,1) to AR(1), a lower-side control chart applicable.

The recommendation for the choice of the CUSUM chart parameter is to select $\delta$ as the half of the shift one is eager to detect. As in our design the absolute size of shifts in $\theta$ in both directions is 1/2, we select the CUSUM parameter $\delta =1/4$ in the Monte Carlo simulations. Concerning the choice of the EWMA smoothing parameter $\lambda$ we consider different values from the interval [0.01, 1], as it is recommended by Lazariv et al. (2015) because even smaller $\lambda$ values could lead to numerical instabilities. Note that all these parameter values are common advices from the literature, the optimal choice of control chart parameters depends on the selected performance measure, the distribution of the control statistic as well as on the size of the actual shift which is (typically) unknown in applications.

Unfortunately, the exact distribution of our control statistic is unknown so we cannot provide theoretical control limits even for the simplest no-memory control charts. In general, for memory control charts (CUSUM and EWMA) it is not possible to get exact theoretical control limits, so that numerical methods should be applied. The path of the process $\{x_t\}$ is simulated $B=10^6$ times, then the process $\{v_t\}$ is computed as in (8). For a given control chart, we obtain the run lengths $RL^{(b)}$ for the repetitions $b=1,...,B$, so the Average Run Length (ARL) is estimated by

$$\begin{aligned} \widehat{ARL}=(1/B) \, \cdot \, \sum _{b=1}^B RL^{(b)}. \end{aligned}$$

In order to get control limits for the control charts, we first simulate the process without any change and compute control limits which provide the in-control ARL equal to $ARL_0=100$. Afterwards, we evaluate the detecting ability of the charts under considerations by comparing them with respect to the out-of-control ARLs denoted by $ARL_1$ where the changes in the MA(1) parameter occurs immediately at $t=1$, which is also known as the zero-state ARL (cf. Montgomery 2013). Moreover, we report the out-of-control conditional steady state (ss) ARL (cf. Knoth 2021) which is defined as $ssARL={{\,\mathrm{E}\,}}(RL-m+1|RL\ge m)$ for a change at $m>1$, i.e., no false alarms until time point *m*. In our application we select $m=51$; of course, the choice $m=1$ corresponds to the zero-state ARL. The results of the Monte Carlo simulations are summarized in Table 1.

**Table 1:** In- and out-of-control ARLs (zero-state $ARL_1$ and conditional steady-state *ssARL*) for detection of changes in MA(1) parameter, Monte Carlo results based on $10^6$ replications, in control $ARL_0=100$

Detecting change from ARMA(1,1) to AR(1) in control ARMA (1,1), $\theta _0=-0.5$, $\theta _1=0$, CUSUM with FIR, $\delta =1/4$: $ARL_1=$** 12.83**, $ssARL=$ ***16.05***										
EWMA $\lambda \in (0,1]:$	0.01	0.02	0.05	0.10	0.25	0.40	0.55	0.70	0.85	1.00
$ARL_0$, $\theta _0=-0.5$	100.23	100.11	100.31	100.07	99.89	99.88	99.96	100.00	99.97	100.09
Zero-state $ARL_1$, $\theta _1=0$	**7.06**	9.20	12.11	13.69	16.32	19.11	22.01	25.37	30.00	36.96
*ssARL*, $\theta _1=0$	25.94	22.41	17.84	***15.94***	17.03	19.52	22.27	25.48	29.90	36.60

Detecting change from AR(1) to ARMA(1,1)										
in control AR(1), $\theta _0=0$, $\theta _1=\,-0.5$, CUSUM with FIR, $\delta =1/4$: $ARL_1=\,$ **14.60**, $ssARL=$ ***17.35***										
EWMA $\lambda \in (0,1]:$	0.01	0.02	0.05	0.10	0.25	0.40	0.55	0.70	0.85	1.00
$ARL_0$, $\theta _0=0$	100.42	100.28	100.17	100.08	99.84	99.97	99.95	100.13	99.85	100.14
$ARL_1$, $\theta _1=-0.5$	**6.77**	8.47	11.15	13.38	16.55	18.22	19.31	20.23	21.14	22.39
*ssARL*, $\theta _1=-0.5$	19.77	18.33	16.37	***16.02***	17.34	18.49	19.36	20.17	21.04	22.27

The smallest value of zero-state $ARL_1$ are marked in bold, those of steady-state $ssARL$ are marked in bold italic, each for CUSUM and EWMA charts

The speed of detecting changes in the MA(1) parameter is measured by out-of-control ARLs. The results in Table 1 indicate that the CUSUM chart detects changes rather slowly compared to the in-control ARL 100, namely the out-of-control (zero-state) ARL is 12.83 for the shift from ARMA(1,1) to AR(1) and 14.60 for the shift from AR(1) to ARMA(1,1). The no-memory Shewhart control charts (i.e., the EWMA charts with $\lambda =1$) are even worse with the out-of-control ARLs 36.96 and 22.39, respectively. Applying the EWMA charts with smaller $\lambda$-values leads to substantial decrease in the out-of-control ARLs. The conventional choice $\lambda =0.1$ provides the out-of-control ARLs 13.69 and 13.38 which are comparable with those from the CUSUM charts. However, a further decrease in $\lambda$ to the value $\lambda =0.01$ provides 7.06 and 6.77, respectively, which are almost twice lower than the out-of-control ARLs for the corresponding CUSUM charts. Hence, the CUSUM monitoring schemes are clearly not optimal for our setting with respect to the out-of-control ARL criterion. Note that a further reduction of $\lambda$ could lead to numerical problems as it is discussed by Lazariv et al. (2015) and Morais et al. (2015).

Concerning the results for the conditional steady-state *ssARL* it is worth to note that $ssARL>ARL_1$ for the small values of $\lambda$, moreover, the smallest *ssARL* are achieved for $\lambda =\,0.1$ (15.94) for the shift to AR(1) and for $\lambda =0.1$ (16.02) for the shift to ARMA(1,1). For $\lambda =\,1$ (i.e., the no-memory Shewhart chart) we observe that $ssARL\approx ARL_1$ which also holds for $\lambda =\,0.7$ and $\lambda =\,0.85$. Although *ssARL*s for the CUSUM are larger than $ARL_1$s, the former get more comparable with the smallers *ssARL*s for the EWMA charts. Hence, for the steady-state ARL criterion the CUSUM chart remains a sound alternative to the EWMA procedures. These detection results are not surprising as we are focused on the detection of quite small shifts compared to the in-control variance of the underlying (monitored) process, see Proposition 2.

## The empirical application

We apply the control charts for monitoring changes both from AR(1) to ARMA(1,1) and from ARMA(1,1) to AR(1) for the series of daily log realized volatility measures. The family of nonlinear GARCH models (cf. Tsay 2010) are typically applied to daily asset returns on financial risky assets in order to assess conditional heteroskedasticity and volatility clustering phenomenon. Time series models which exploit realized volatility measures based on intraday (high-frequency) information serve to the same purpose. Such time series models [e.g., those of Corsi (2009) and its further modifications] are widely used nowadays as realized volatilities are believed to be more precise measures of the true volatility compared to squared daily returns which are exploited in GARCH-type models.

Realized volatilities are consistent estimators of the true daily integrated volatilities under fairly general regularity conditions (cf. Barndorff-Nielsen and Shephard 2002). Hence, realized volatility measures would converge in probability to the true daily integrated volatilities when the number of intraday observations is very large. Empirically, however, measurement errors appear be present in realized volatility series as the number of observations per day is finite. The daily volatility model with measurement errors in realized volatilities is discussed e.g., by Bollerslev et al. (2016) in their Section 2.2. In particular, they argue “The consistency of realized volatility for integrated volatility estimation, coupled with the fact that the measurement error is serially uncorrelated under general conditions, motivate the use of reduced form time series models for the observable realized volatility as a simple way to forecast the latent integrated volatility of interest.” It is much related to our setting, in particular, the AR(1) dynamics of the state variables corresponds to Eq. (6) in Bollerslev et al. (2016) and originates—to the best of our knowledge—from the stochastic volatility literature (cf. Jacquir et al. 1994).

The log transformation of realized volatilities is a commonly applied procedure for their time series modeling as it provides a distribution which is more symmetric and closer to normality (cf. Andersen et al. 2001). Hence, we extend the online monitoring approach of Golosnoy et al. (2012) where the aim is to detect changes in the mean of an ARMA(1,1) model for daily log realized volatilities.

We consider daily realized volatility time series of three important stock market indices S&P500 (SPX), DAX 30 (GDAXI), and Nikkei 225 (N225) which are obtained from the Oxford-Man Institute’s realized library (cf. Heber et al. 2009). The data covers the period 2002–2019. For estimation purposes we consider the observations in 2002–2006 as the in-sample period which is a (comparatively) ‘calm’ time period on financial markets before the start of the financial crisis in 2007, whereas the remaining years 2007–2019 form the out-of-sample period. Hence, our sample ends before the outbreak of Covid-19 pandemic situation which has dramatically influenced the financial markets world-wide at the beginning of 2020.

We represent daily log realized volatilities denoted by $RV_t$ by means of a multiplicative volatility component model (cf. Engle and Sokalska 2012) which has a convenient property to be additive in logs:

15 $$\begin{aligned} RV_t=\widetilde{RV}_t + y_t, \end{aligned}$$

where $\widetilde{RV}_t$ is the long-term (possibly non-stationary) component and $y_t$ is the remaining short-memory part which is of our interest. Next, we filter out the long-term component $\widetilde{RV}_t$ by the exponential smoothing

16 $$\begin{aligned} {\widetilde{RV}}_t= \gamma RV_{t-1} + (1-\gamma )\widetilde{RV}_{t-1}, \qquad \text{ with } \quad \gamma =0.05, \end{aligned}$$

in order to extract the short-term component $y_t$. The value $\gamma =0.05$ reflects the impact of new information and is the empirically recommended value for the exponential smoothing approach (cf. Golosnoy et al., 2019). For the initialization, we set $\widetilde{RV}_0$ equal to the long-run average of $RV_t$, so the mean of $y_t$ is zero.

For the extracted process $\{y_t\}$ we estimate by the maximum likelihood approach both AR(1) or ARMA(1,1) models based on the in-sample period 2002–2006, as well as based on the full sample. We report the estimates in Table 2 together with the AIC and BIC goodness-of-fit measures. Note that all series are clearly stationary. Based on the AIC/BIC criteria, the ARMA(1,1) is preferred over AR(1) for all three time series which indicates on the presence of measurement errors, in line with the findings of Bollerslev et al. (2016). The parameter estimates from the in-sample period are very similar to those obtained from the full sample which is an indicator that the in-sample period provides a good representation of the complete sample.

**Table 2:** Parameter estimates for AR(1) and ARMA(1,1) models for $y_t$-processes

Index, model	$\widehat{\phi }$	$\widehat{\theta }$	$\widehat{\sigma }^2_a$	AIC/$10^3$	BIC/$10^3$
In sample parameter estimates, 2002–2006					
S&P500, AR(1)	0.556	–	0.420	6.441	6.454
S&P500, ARMA(1,1)	0.818	– 0.401	0.398	6.271	6.289
DAX30, AR(1)	0.485	–	0.297	5.358	5.370
DAX30, ARMA(1,1)	0.827	– 0.479	0.281	5.175	5.194
Nikkei225, AR(1)	0.510	–	0.334	5.542	5.554
Nikkei225, ARMA(1,1)	0.795	– 0.405	0.320	5.406	5.424

Full sample parameter estimates, 2002-2019					
S&P500, AR(1)	0.522	–	0.378	8.434	8.447
S&P500, ARMA(1,1)	0.824	− 0.441	0.357	8.175	8.195
DAX30, AR(1)	0.467	–	0.291	7.328	7.341
DAX30, ARMA(1,1)	0.828	$-0.497$	0.276	7.089	7.109
Nikkei225, AR(1)	0.500	–	0.302	7.211	7.224
Nikkei225, ARMA(1,1)	0.796	$-0.419$	0.289	7.031	7.050

All estimates are statistically different from zero at 1% level

Then we compute $x_t$ and $v_t$ series as discussed in Sect. 2 for either AR(1) or ARMA(1,1) model estimates both in sample and out of sample. Based on the process $\{v_t\}$, we apply the CUSUM and EWMA control charts presented in Sect. 3. The normality assumption used in the Monte Carlo simulations is usually rejected by empirical modeling of log realized volatilities (cf. Golosnoy et al. 2012). This happens in many situations due to outliers which lead to undesired false signals. In order to reduce the number of false signals in the empirical study we select the control limits such that they provide a more conservative in-control ARL value 200 which corresponds to around 16 false signals for the in-control state for the monitoring period 2007–2019 with ca. 3300 daily observations. We apply both upper and lower control charts for monitoring ARMA(1,1) processes, whereby for monitoring AR(1) processes we apply only the lower-sided charts. For the EWMA charts we set the smoothing parameter $\lambda =0.01$. For the lower-sided CUSUM for the in-control AR(1) we set $\delta =1/4$ as the half of change from $\theta _0=0$ to $\theta _1=-1/2$, see (12). For the CUSUM charts with the in-control ARMA(1,1) we select the parameter $\delta$ in a data-driven way. In particular, for the estimated ARMA(1,1) parameters $\hat{\phi }$ and $\hat{\theta }$ we choose $\delta =-\hat{\theta }/2$ for the upper CUSUM charts for detection of changes from AR(1) to ARMA(1,1) and set $\delta =(\hat{\theta }-\hat{\phi })/2$ for the lower CUSUM charts for detection of changes in $\theta$ to the direction further away from AR(1) to the boundary value $-\phi$. Given AR(1) or ARMA(1,1) estimates obtained either from in sample or from full sample, and the pre-selected parameters of the control charts, we compute the control limits for the in-control state by a Monte Carlo simulation with $10^6$ replications. Then we apply the control charts by making a re-start after each obtained signal.

**Table 3:** Number of CUSUM and EWMA signals, in control ARL=200 with 16 in-control signals expected

Number of signals for reject of AR(1)				
In control AR(1), change to ARMA(1,1)-direction	In sample estimates		Full sample estimates	
Lower control charts, $-\hat{\phi }<\theta _1<\theta _0=0$	EWMA	CUSUM	EWMA	CUSUM
S&P500	100	60	87	68
DAX30	69	47	62	44
Nikkei225	70	42	72	49

Number of signals for reject of ARMA(1,1)				
In control ARMA(1,1), change to AR(1)-direction	In sample estimates		Full sample estimates	
Upper control charts, $0\ge \theta _1>\theta _0=\hat{\theta }$	EWMA	CUSUM	EWMA	CUSUM
S&P500	31	28	31	37
DAX30	29	29	25	33
Nikkei225	19	20	17	23

In control ARMA(1,1), change further away from AR(1)	In sample estimates		Full sample estimates	
Lower control charts, $-\hat{\phi }<\theta _1<\theta _0=\hat{\theta }<0$	EWMA	CUSUM	EWMA	CUSUM
S&P500	37	27	44	30
DAX30	20	20	18	19
Nikkei225	15	19	22	24

In Table 3 we provide the number of empirical reject signals for monitoring both AR(1) and ARMA(1,1) models. Concerning monitoring validity of the AR(1) model in Table 3 (the upper block), the number of signals from the EWMA charts is much higher than from the CUSUM charts for all three financial markets, as it is also expected from our Monte Carlo results. The obtained number of signals for S&P500 index is much higher than for DAX 30 and Nikkei 225. Remarkably, there is no much difference for the number of signals for the in sample and full sample parameter estimates. Note that our results based on the full-sample estimate provide a kind of Phase I analysis for the period 2007-2019. In general, the number of obtained signals from the CUSUM charts is at least three times higher than it can be expected in control. This indicates on the evidence that the simple AR(1) approach is not sufficient for the modeling purposes of the short-run component of realized volatility and the addition of the MA(1) component could be useful.

The number of signals by monitoring validity of the ARMA(1,1) models in Table 3 (the lower block) is more balanced for the CUSUM and EWMA charts. The rejects of ARMA(1,1) by the upper control charts are the support for a change in the direction of AR(1) alternative, whereas the rejects of ARMA(1,1) by the lower charts are in favor of a more pronounced MA(1) component. As it is to expect, the number of signals from the lower charts for monitoring ARMA(1,1) is in all cases smaller than from the lower charts for AR(1) monitoring. Concerning the upper control charts, there are about twice more signals than expected for S&P500 and DAX30, whereas the number of signals for Nikkei225 roughly corresponds to the in-control expectations. This points on the evidence that ARMA(1,1) provides a fair time series modeling for Nikkei225 during the considered period of time.

**Fig. 1:**
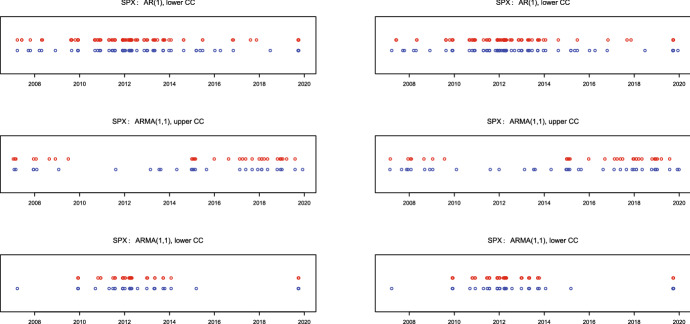
Signals for S&P500: EWMA (red), CUSUM (blue) with charts based on in sample (left) and full sample (right) parameter estimates (colour figure online)

**Fig. 2:**
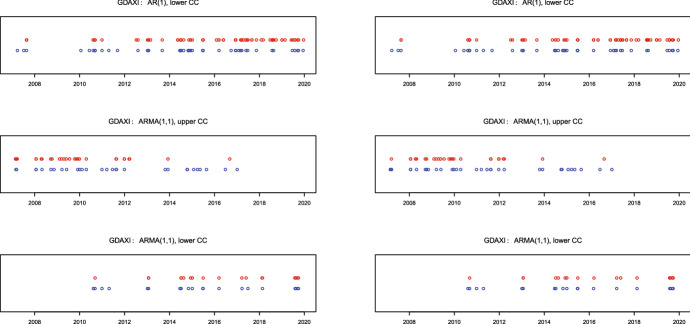
Signals for DAX 30: EWMA (red), CUSUM (blue) with charts based on in sample (left) and full sample (right) parameter estimates (colour figure online)

**Fig. 3:**
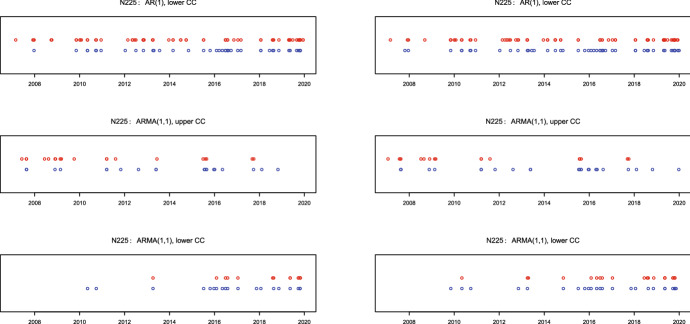
Signals for Nikkei 225: EWMA (red), CUSUM (blue) with charts based on in sample (left) and full sample (right) parameter estimates (colour figure online)

To illustrate the signal timings we provide in Figs. 1, 2, and 3 the dates of the control charts alarms for S&P500, DAX 30, and Nikkei 225, respectively. EWMA signals are depicted as red points, whereas CUSUM signals as blue points. The upper plots show the signals for in-control AR(1) and out-of-control ARMA(1,1), the middle plots for the in-control ARMA(1,1) and out-of-control changes in AR(1) direction, whereas the lower plots the in-control ARMA(1,1) and out-of-control in the direction of a more pronounced ARMA(1,1) model with an MA(1) parameter closer than in control to the boundary value $-\hat{\phi }$. Hence, in the upper plots we confront the rejects of AR(1) whereas in the middle and lower plots the rejects of ARMA(1,1) models.

We observe for all panels that the timing of signals from the CUSUM and EWMA charts coincide to much extent which is an indicator that both charts do react on possible shifts in the MA(1) part. Moreover, it is clearly observable that signals from all charts are clustered in time. In particular, signals from the upper charts (the middle panel) for a less pronounced MA(1) component are mostly located during the subprime mortgage crisis in 2007–2009. This is not surprising, as it is well-known that the speed of information transfer (related to the number of news per day) increases substantially during crisis periods, so that the MA(1) component gets superfluous in such situations. On the contrary, the lower charts for a more pronounced MA(1) component provide signals mostly in the (comparatively) calm post-crises period starting from 2011. Since the speed of information transfer usually decreases during the calm time, this finding also appears to be rather plausible.

In general, it is not always an easy task to differentiate between correct and false (i.e., driven by outliers) signals. Although standing alone outliers could trigger false signals by control charts, note that for our statistic (8) based on the product $x_tx_{t-1}$ their impact would be mitigated compared with (for example) a statistic based on the square $x^2_t$. In our case an outlier $x_t$ would influence both $x_tx_{t-1}$ and $x_{t+1}x_t$, so that in the empirical application we could then expect some separately standing back-to-back (pairs of) signals depicted as strongly overlapping circles. We indeed observe some of such alone-standing pairs for all considered markets, however, the majority of signals are not alone-standing but clustered in time indicating on structural changes. For this reason we suppose that the outlier problem is not pronounced much in our application.

Finally, we would like to discuss some possible directions for future research. The first possibility is to exploit more fully the available high-frequency information by monitoring the MEM model estimated based (say) on hourly data. In this setting, however, one should take into account the stylized facts which are typical for intraday asset returns, for example the intraday periodicity issue (cf. Dette et al. 2022a, b) and the related problems. The second possible direction for further research is related to the economic interpretation of signals and transferring them into financial decisions. To this extent note that in general signals from control charts in financial applications are more a supplementary decision-making tool which indicates on the necessity of the further in-depth analysis. This is much different from situations in macroeconomic applications as e.g., by sequential monitoring of the business cycle (cf. Golosnoy and Hogrefe 2013) or monitoring changes in the inflation expectation process (cf. Golosnoy and Roestel 2019) where—based on publicly available information—the majority of signals could be (although often with some time delay) either interpreted as reasonable ones or classified as outliers. On the contrary, interpreting dates of financial signals is not an easy task, as it would require gathering much more external information which appears to be a costly process. For this reason, this would go beyond the illustration of our methodology in the current paper but could be an interesting topic for future investigations. Finally, the monitoring of MEM models could be extended for monitoring MEMs in multivariate settings (cf. Golosnoy and Rossen 2018) or for the purpose of monitoring for changes in various types of networks (cf. Klüppelberg and Seifert 2019, 2020; Chen et al. 2022).

## Summary

We consider a measurement error model (MEM) where the state variable follows AR(1) dynamics. Such a MEM could be equivalently written as an ARMA(1,1) model, however, when the measurement error gets negligible the MEM reduces to a simple AR(1) representation. We focus on the task of sequential monitoring of the MEM which is equivalent to monitoring the MA(1) parameter with the aim to detect possible changes in the MA(1) part as soon as they occur. For this purpose we elaborate both CUSUM and EWMA control charts for online detection of changes from AR(1) to ARMA(1,1) and vice versa. The control statistic for these charts is based on the testing approach elaborated by Golosnoy et al. (2021). In the Monte Carlo simulation study we show that these changes (which are numerically rather small compared to the process variance) could be detected not immediately but only with some detection delay. The empirical illustration is based on monitoring of AR(1) and ARMA(1,1) models for the short-run component of daily log realized volatility series of the leading stock market indices. The obtained signals support the evidence that the AR(1) models are more suitable during crisis periods, whereas the MEM, i.e., the ARMA(1,1) representation, for no-crisis periods in financial markets.

## Appendix

### Proof of Proposition 1

We provide the expressions for $\theta$ and $\sigma _a^2$ of the ARMA(1,1) model as the functions of the MEM parameters $\phi , \sigma _{\epsilon }^2$ and $\sigma _u^2$ in order to show that MEM and ARMA(1,1) model are equivalent in distributions. Consider $x_t=y_t-\phi y_{t-1}$ for both formulations:

$$ \begin{array}{*{20}c}    {{\text{MEM}}:} & {y_{t}  - \phi y_{{t - 1}}  = \varepsilon _{t}  + u_{t}  - \phi u_{{t - 1}} ,}  \\    {{\text{ARMA(1,1):}}} & {y_{t}  - \phi y_{{t - 1}}  = a_{t}  + \theta a_{{t - 1}} .}  \\   \end{array}  $$

In order to show the equivalence, we consider the autocovariances for both representations

17 $$\begin{aligned} {{\,\mathrm{Var}\,}}(x_t)= & {} {{\,\mathrm{E}\,}}(x^2_t) = \sigma ^2_{\epsilon }+\sigma ^2_u(1+\phi ^2)\overset{!}{=} \sigma ^2_a(1+\theta ^2)\nonumber \\ {{\,\mathrm{Cov}\,}}(x_t,x_{t-1})= & {} {{\,\mathrm{E}\,}}(x_t x_{t-1})=-\phi \sigma ^2_u\overset{!}{=} \theta \sigma ^2_a. \end{aligned}$$

From the second equation we get

$$\begin{aligned} \sigma ^2_a= -\frac{\phi }{\theta } \sigma ^2_u, \end{aligned}$$

which implies that $\theta$ has an opposite sign than $\phi$. Substituting it into the first equation yields

$$\begin{aligned} 0=\, & {} \sigma _{\epsilon }^2 + \sigma _u^2 (1+\phi ^2)+(\theta ^{-1} + \theta )\phi \sigma _u^2,\\ \quad \Leftrightarrow \quad 0=\, & {} \theta ^2 + \frac{\sigma _{\epsilon }^2 + \sigma _u^2 (1+\phi ^2)}{\phi \sigma _u^2}\theta +1. \end{aligned}$$

Solving this quadratic equation yields two possible solutions

18 $$\begin{aligned} \theta _{1|2}=- \frac{\sigma ^2_{\epsilon }+\sigma ^2_u(1+\phi ^2)}{2\phi \sigma ^2_u} \pm \sqrt{\left[ \frac{\sigma ^2_{\epsilon }+\sigma ^2_u(1+\phi ^2)}{2\phi \sigma ^2_u}\right] ^2-1}. \end{aligned}$$

The radicand in (18) is positive, because $\sigma ^2_{\epsilon }+\sigma ^2_u(1-\phi )^2>0$ implies that $\sigma ^2_{\epsilon }+\sigma ^2_u(1+\phi ^2)>2\phi \sigma ^2_u$.

As we focus on $\phi \in (0,1)$ we get the unique solution:

19 $$\begin{aligned} \theta =- \frac{\sigma ^2_{\epsilon }+\sigma ^2_u(1+\phi ^2)}{2\phi \sigma ^2_u} + \sqrt{\left[ \frac{\sigma ^2_{\epsilon }+\sigma ^2_u(1+\phi ^2)}{2\phi \sigma ^2_u}\right] ^2-1} \, =:\, -b+\sqrt{b^2-1}\,, \end{aligned}$$

as it guarantees that $\theta \in (-1,0)$ as $b>1$ implies that $b^2-1 >(b-1)^2$ and, hence, $\theta =-b+\sqrt{b^2-1}>-1$. Note that the other solution leads to $\theta <-1$ in the MA(1) part of the ARMA(1,1). $\square$

### Proof of Proposition 2

From Eq. (17) we get that ${{\,\mathrm{E}\,}}(x_t x_{t-1})= \theta \sigma ^2_a$ which yields ${{\,\mathrm{E}\,}}(v_t)=\theta$. We can write

$$\begin{aligned} \sigma ^2_a v_t=x_tx_{t-1}=(a_t+\theta a_{t-1})(a_{t-1}+\theta a_{t-2}) =a_ta_{t-1}+\theta a_ta_{t-2} +\theta a_{t-1}^2+\theta ^2a_{t-1}a_{t-2}. \end{aligned}$$

Next we observe that

$$ \begin{gathered}   \sigma^{4} _{a} v_{t}^{2}  = a_{t}^{2} a_{{t - 1}}^{2}  + \theta a_{t}^{2} a_{{t - 1}} a_{{t - 2}}  + \theta a_{t} a_{{t - 1}}^{3}  + \theta ^{2} a_{t} a_{{t - 1}}^{2} a_{{t - 2}}  \hfill \\   \quad \quad \, + \theta a_{t}^{2} a_{{t - 1}} a_{{t - 2}}  + \theta ^{2} a_{t}^{2} a_{{t - 2}}^{2}  + \theta ^{2} a_{t} a_{{t - 1}}^{2} a_{{t - 2}}  + \theta ^{3} a_{t} a_{{t - 1}} a_{{t - 2}}^{2}  \hfill \\   \quad \quad \, + \theta a_{t} a_{{t - 1}}^{3}  + \theta ^{2} a_{t} a_{{t - 1}}^{2} a_{{t - 2}}  + \theta ^{2} a_{{t - 1}}^{4}  + \theta ^{3} a_{{t - 1}}^{3} a_{{t - 2}}  \hfill \\   \quad \quad \, + \theta ^{2} a_{t} a_{{t - 1}}^{2} a_{{t - 2}}  + \theta ^{3} a_{t} a_{{t - 1}} a_{{t - 2}}^{2}  + \theta ^{3} a_{{t - 1}}^{3} a_{{t - 2}}  + \theta ^{4} a_{{t - 1}}^{2} a_{{t - 2}}^{2} . \hfill \\  \end{gathered}  $$

Then we immediately obtain ${{\,\mathrm{E}\,}}(v_t^2)=1+4\theta ^2+\theta ^4$ and ${{\,\mathrm{Var}\,}}(v_t)= 1+3\theta ^2+\theta ^4$ because the fourth moment of $a_t \sim \, \mathcal {N}_{\text{ iid }}(0,\sigma ^2_a)$ is $3\sigma ^4_a$. Now consider

$$ \begin{gathered}   \sigma^{4}_{a} v_{t} v_{{t - 1}}  = a_{t} a_{{t - 1}}^{2} a_{{t - 2}}  + \theta a_{t} a_{{t - 1}}^{2} a_{{t - 3}}  + \theta a_{t} a_{{t - 1}} a_{{t - 2}}^{2}  + \theta ^{2} a_{t} a_{{t - 1}} a_{{t - 2}} a_{{t - 3}}  \hfill \\   \quad \quad \quad \;\, + \theta a_{t} a_{{t - 1}} a_{{t - 2}}^{2}  + \theta ^{2} a_{t} a_{{t - 1}} a_{{t - 2}} a_{{t - 3}}  + \theta ^{2} a_{t} a_{{t - 2}}^{3}  + \theta ^{3} a_{t} a_{{t - 2}}^{2} a_{{t - 3}}  \hfill \\   \quad \quad \quad \;\, + \theta a_{{t - 1}}^{3} a_{{t - 2}}  + \theta ^{2} a_{{t - 1}}^{3} a_{{t - 3}}  + \theta ^{2} a_{{t - 1}}^{2} a_{{t - 2}}^{2}  + \theta ^{3} a_{{t - 1}}^{2} a_{{t - 2}} a_{{t - 3}}  \hfill \\   \quad \quad \quad \;\, + \theta ^{2} a_{{t - 1}}^{2} a_{{t - 2}}^{2}  + \theta ^{3} a_{{t - 1}}^{2} a_{{t - 2}} a_{{t - 3}}  + \theta ^{3} a_{{t - 1}} a_{{t - 2}}^{3}  + \theta ^{4} a_{{t - 1}} a_{{t - 2}}^{2} a_{{t - 3}} , \hfill \\  \end{gathered}  $$

which gives that ${{\,\mathrm{E}\,}}(v_tv_{t-1})=2\theta ^2$ and ${{\,\mathrm{Cov}\,}}(v_t,v_{t-1})=\theta ^2$. Finally, for $\ell \ge 2$ we obtain

$$\begin{aligned}\sigma^4_a v_t v_{t-\ell}= a_ta_{t-1}a_{t-\ell}a_{t-\ell-1} + \theta a_ta_{t-1}a_{t-\ell}a_{t-\ell-2}\\ \quad + \theta a_ta_{t-1}a_{t-\ell-1}^2 + \theta^2 a_ta_{t-1}a_{t-\ell-1}a_{t-\ell-2}\\ \quad +\theta a_ta_{t-2}a_{t-\ell}a_{t-\ell-1} +\theta^2 a_ta_{t-2}a_{t-\ell}a_{t-\ell-2} +\theta^2 a_ta_{t- 2}a^2_{t-\ell-1}\\\quad +\theta^3 a_ta_{t-2}a_{t-\ell-1}a_{t-\ell-2} \\ \quad + \theta a^2_{t-1}a_{t-\ell}a_{t-\ell-1} + \theta^2 a^2_{t-1}a_{t-\ell}a_{t-\ell-2} + \theta^2 a^2_{t-
1}a^2_{t-\ell-1} + \theta^3a^2_{t-1}a_{t-\ell-1}a_{t-\ell-2} \\ \quad + \theta^2 a_{t-1}a_{t-2}a_{t-\ell}a_{t-\ell-1} + \theta^3 a_{t-1}a_{t-2}a_{t-\ell}a_{t-\ell-2} \\ \quad + \theta^3 a_{t-1}a_{t-2}a^2_{t-\ell-1} + \theta^4 a_{t-1}a_{t-2}a_{t-\ell-1}a_{t-\ell-2}.
 \end{aligned}$$

This leads to ${{\,\mathrm{E}\,}}(v_tv_{t-\ell })=\theta ^2$ and, consequently, to ${{\,\mathrm{Cov}\,}}(v_t,v_{t-\ell })=0$ for $\ell \ge 2$. $\square$

## References

[CR1] Andersen TG, Bollerslev T, Diebold FX, Ebens H (2001). The distribution of realized stock return volatility. J. Financ. Econ..

[CR2] Barndorff-Nielsen O, Shephard N (2002). Econometric analysis of realized volatility and its use in estimating stochastic volatility models. J. Roy. Stat. Soc. B.

[CR3] Bodnar O, Schmid W (2007). Surveillance of the mean behaviour of multivariate time series. Stat. Neerl..

[CR4] Bollerslev T, Patton AJ, Quaedvlieg R (2016). Exploiting the errors: a simple approach for improved volatility forecasting. J. Econ..

[CR5] Brockwell PJ, Davis RA (2009). Time Series: Theory and Methods.

[CR6] Chen CY-H, Okhrin Y, Wang T (2022). Monitoring network changes in social media. J. Bus. Econ. Stat..

[CR7] Corsi F (2009). A simple approximative long-memory model of realized volatility. J. Financ. Economet..

[CR8] Dette H, Golosnoy V, Kellermann J (2022). Correcting intraday periodicity bias in realized volatility measures. Econ. Stat..

[CR9] Dette H, Golosnoy V, Kellermann J (2022). The effect of intraday periodicity on realized volatility measures. Metrika.

[CR10] Durbin J, Koopman SJ (2009). Time Series Analysis by State Space Methods.

[CR11] Engle RF, Sokalska M (2012). Forecasting intraday volatility in the US equity market. Multiplicative component GARCH. J. Financ. Economet..

[CR12] Golosnoy V, Okhrin I, Schmid W (2012). Statistical surveillance of volatility forecasting models. J. Financ. Economet..

[CR13] Golosnoy V, Hogrefe J (2013). Signaling NBER turning points: a sequential approach. J. Appl. Stat..

[CR14] Golosnoy V, Gribisch B, Seifert MI (2019). Exponential smoothing of realized portfolio weights. J. Empir. Financ..

[CR15] Golosnoy V, Roestel J (2019). Real time monitoring of the US inflation expectation process. Macroecon. Dyn..

[CR16] Golosnoy V, Rossen A (2018). Modeling dynamics of metal price series via state space approach with two common factors. Empir. Econ..

[CR17] Golosnoy V, Köhler S, Schmid W, Seifert MI (2021). Testing for parameter changes in linear state space models. Appl. Stoch. Mod. Bus. Ind..

[CR18] Golosnoy V, Seifert M.I (2021). Online monitoring of mean changes in high-dimensional persistent linear time series. Stat.: J. Theor. Appl. Stat..

[CR19] Hamilton, J.D.: State-space models. In Handbook of Ecoonometrics, eds. R.F. Engle and D.L. McFadden, vol. 4, chapter 50: 3014–3077 (1994a)

[CR20] Hamilton JD (1994). Time Series Analysis.

[CR21] Heber, G., Lunde, A., Shephard, N., Sheppard, K.: Oxford–Man Institute’s realized library (v0.3), Oxford-Man Institute, University of Oxford (2009)

[CR22] Jacquier E, Polson NG, Rossi P (1994). Bayesian analysis of stochastic volatility models (with discussion). J. Bus. Econ. Stat..

[CR23] Jiang W, Tsui KL, Woodall WH (2000). A new SPC monitoring method: the ARMA chart. Technometrics.

[CR24] Kim CJ, Nelson CR (1999). State Space Models with Regime Switching.

[CR25] Knoth S (2021). Steady-state average run length(s): methodology, formulas, and numerics. Seq. Anal..

[CR26] Klüppelberg C, Seifert MI (2019). Financial risk measures for a network of individual agents holding portfolios of light-tailed objects. Fin. Stochast..

[CR27] Klüppelberg C, Seifert MI (2020). Explicit results on conditional distributions of generalized exponential mixtures. J. Appl. Probab..

[CR28] Lazariv T, Okhrin Y, Schmid W (2015). Behavior of EWMA type control charts for small smoothing parameters. Comput. Stat. Data Anal..

[CR29] Lazariv T, Schmid W (2019). Surveillance of non-stationary processes. AStA Adv. Stat. Anal..

[CR30] Lu C-W, Reynolds MR (2001). CUSUM charts for monitoring an autocorrelated process. J. Qual. Technol..

[CR31] Lucas JM, Crosier RB (1982). Fast initial response for CUSUM quality control schemes. Technometrics.

[CR32] Montgomery DC (2013). Statistical Quality Control: A Modern Introduction.

[CR33] Morais MC, Okhrin Y, Schmid W (2015). Quality surveillance with EWMA control charts based on exact control limits. Stat. Pap..

[CR34] Okhrin Y, Schmid W, Surveillance Financial (2008). Surveillance of univariate and multivariate linear time series. Frisén, M.

[CR35] Rabyk L, Schmid W (2016). EWMA control charts for detecting changes in the mean of a long-memory process. Metrika.

[CR36] Rosolowski M, Schmid W (2006). EWMA control charts for monitoring the mean and the autocovariances of stationary processes. Stat. Pap..

[CR37] Schmid W (1997). CUSUM control schemes for Gaussian processes. Stat. Pap..

[CR38] Tsay RS (2010). Analysis of Financial Time Series.

